# Promoter DNA Methylation Regulates Murine SUR1 (*Abcc8*) and SUR2 (*Abcc9*) Expression in HL-1 Cardiomyocytes

**DOI:** 10.1371/journal.pone.0041533

**Published:** 2012-07-23

**Authors:** Naheed Fatima, James F. Schooley, Willliam C. Claycomb, Thomas P. Flagg

**Affiliations:** 1 Department of Anatomy, Physiology and Genetics, Uniformed Services University of the Health Sciences, F. Edward Hebert School of Medicine, Bethesda, Maryland, United States of America; 2 Department of Biochemistry and Molecular Biology, LSU Health Sciences Center, New Orleans, Louisiana, United States of America; Peking University Health Science Center, China

## Abstract

Two mammalian genes encode the SURx (SUR1, *Abcc8* and SUR2, *Abcc9*) subunits that combine with Kir6.2 (*Kcnj11*) subunits to form the ATP-sensitive potassium (KATP) channel in cardiac myocytes. Different isoform combinations endow the channel with distinct physiological and pharmacological properties, and we have recently reported that the molecular composition of sarcolemmal KATP channels is chamber specific in the mouse heart. KATP channel composition is determined by what subunits are expressed in a cell or tissue. In the present study, we explore the role of CpG methylation in regulating SUR1 and SUR2 expression. In HL-1 cardiomyocytes, as in atrial myocytes, SUR1 expression is markedly greater than SUR2. Consistent with CpG methylation-dependent silencing of SUR2 expression, bisulfite sequencing of genomic DNA isolated from HL-1 cells demonstrates that 57.6% of the CpGs in the promoter region of the SUR2 gene are methylated, compared with 0.14% of the the CpG residues in the SUR1 sequence. Moreover, treatment with 10 µM 5-aza-2′-deoxycytidine (Aza-dC) significantly increased both the unmethylated fraction of the SUR2 CpG island and mRNA expression. However, we cannot rule out additional mechanisms of Aza-dC action, as Aza-dC also causes a decrease in SUR1 expression and lower doses of Aza-dC do not alter the unmethylated DNA fraction but do elicit a small increase in SUR2 expression. The conclusion that DNA methylation alone is not the only regulator of SUR subunit expression is also consistent with observations in native myocytes, where the CpG islands of both SUR genes are essentially unmethylated in both atrial and ventricular myocytes. Collectively, these data demonstrate the potential for CpG methylation to regulate SURx subunit expression and raises the possibility that regulated or aberrant CpG methylation might play a role in controlling channel structure and function under different physiological conditions or different species.

## Introduction

ATP-sensitive potassium (K_ATP_) channels are expressed in a diverse set of excitable tissues and provide a direct molecular link between metabolism and function. By responding to changes in the ratio of [ADP] to [ATP] in the cell, K_ATP_ channels modulate cell membrane excitability, controlling Ca^2+^ entry into the cell and Ca^2+^-dependent cell functions. At the molecular level, all K_ATP_ channels share the same general structural blueprint [1]: an inward rectifier potassium channel (Kir6.x) and a sulfonylurea receptor (SURx) coassemble in a 4∶4 stoichiometry to form a single K_ATP_ channel complex. Kir6.x encodes the binding site for inhibitory ATP and forms the conducting pore of the channel. SURx subunit confers sulfonylurea sensitivity to the channel and determines efficacy of potassium channel opening drugs (KCOs) such as diazoxide and pinacidil, and its nucleotide binding folds are essential for nucleotide diphosphate-dependent stimulation [2]–[4]. There are two known genes encoding both the Kir6.x subunits (*Kcnj11;* Kir6.2 and *Kcnj8;* Kir6.1) and SURx subunits (*Abcc8*; SUR1 and *Abcc9;* SUR2) [5]–[8]. Additional combinations are made possible by alternatively spliced isoforms of the SURx subunits [8], [9].

While the same overall architecture is maintained for all K_ATP_ channels, the specific components comprising the channel differ in different tissue types. In pancreatic β-cells, K_ATP_ channels are formed by coassembly of SUR1 and Kir6.2 [6], [10]–[13], while SUR2A joins with Kir6.2 to form the sarcolemmal channel in cardiac ventricular myocytes [10]–[14]. Subunit composition determines, in part, the dynamic range of channel activity. For example, channel complexes containing SUR1 are more sensitive to stimulation by ADP than those containing SUR2A [15], which may underlie the observation that pancreatic β-cell K_ATP_ channels (*SUR1* + Kir6.2) respond to physiological changes in blood glucose, while ventricular K_ATP_ channels (*SUR2A* + Kir6.2) appear less responsive to such small metabolic challenges. This may explain, in part, the observation that transgenic overexpression of an ATP-insensitive Kir6.2 mutant channel (Kir6.2[ΔN30,K185Q]) in the heart has little apparent effect on cardiac function [16], while similar mutants cause profound neonatal diabetes [17], [18], corroborated by more recent observations in transgenic mice expressing Kir6.2V59M, a disease-causing mutation associated with human neonatal diabetes [19], [20].

Expression of each K_ATP_ channel subunit has been reported in the heart and all combinations of subunits have been shown to occur when K_ATP_ channel subunits are coexpressed in heterologous systems [6], [7], [21]–[28]. However, we have recently shown that sarcolemmal K_ATP_ channel composition in the heart is chamber-specific. Atrial channels are made up of Kir6.2 and SUR1, while ventricular channels contain Kir6.2 and SUR2A [29], [30]. Importantly, this structural heterogeneity matches with atrial (SUR1) and ventricular (SUR2) subunit mRNA expression, suggesting that isoform-specific K_ATP_ channel composition is regulated by SURx subunit transcription. Recently it has been reported that an additional combination of subunits (SUR2B and Kir6.1/Kir6.2) comprises the K_ATP_ channel in cells of the conduction system and once again channel composition seems to reflect the subunits that are expressed in those cells [31].

Little is known about the mechanisms that regulate SURx subunit transcription. Deletion analysis of the human or mouse SUR1 genes identified the short sequences (173 bp in human 84 bp in mouse) immediately upstream of exon 1 as the minimal promoter in reporter expression assays carried out in MIN6 and HIT-T15 insulinoma cells [32], [33]. The SUR2 promoter has also been shown to be activated by hypoxia via AP-1 signaling in the H9c2 cell line [34]. Interestingly, the first exon and proximal upstream sequences of the SUR1 and SUR2 genes are GC rich, constituting CpG islands which are potential targets for DNA methylation-dependent regulation of gene expression [35]. In the present study, we explore the role of DNA methylation in regulating the expression of SUR1 and SUR2.

## Materials and Methods

### C57Bl6/J Mice

All expression studies in native tissue were carried out in C57Bl6/J mice (Jackson Laboratories). All mice were male, aged 2–4 months. All procedures complied with the standards for the care and use of animal subjects as stated in the *Guide or the Care and Use of Laboratory Animals* (NIH publication No. 85-23, revised 1996) and protocols were approved by the Institutional Animal Care and Use Committee at the Uniformed Services University of the Health Sciences.

### Cell Culture

Cell cultures were maintained in Claycomb medium (Sigma) supplemented with 10% fetal bovine serum (Sigma, Lot # 8A0177), 0.1 mM norepinephrine, 100 µg/mL penicillin/streptomycin and 0.25 µg/mL amphotericin B. Cells were plated in tissue culture flasks coated overnight with gelatin (0.02% w/v) and fibronectin (0.5% v/v). In experiments to inhibit DNA methylation, cells were incubated in standard culture medium supplemented with 10 µM 5-Aza-2′-deoxycytidine (Aza-dC, Sigma) for 72 hours. Culture medium was changed daily.

### Quantitative RT-PCR

Relative expression of K_ATP_ channel subunit mRNA was examined using quantitative RT-PCR [36]. Briefly, total RNA was isolated from HL-1 cells in culture and from cardiac atrial or ventricular tissue using RNAzol (Fermentas) following manufacturer’s protocols. Isolated RNA was then treated with DNAseI to digest residual genomic DNA and further purified using a silica-based column protocol. RNA concentration was determined spectrophotometrically (Nanodrop Technologies, Inc). cDNA was synthesized from 1 µg RNA (Superscript III, Invitrogen). PCR was carried out using a CFX384 Real Time PCR Detection System (Bio-Rad, Inc.), using Taqman© probe and primer pairs (Applied Biosystems, Inc.) for monitoring reaction progress. 20 ng of template cDNA was used in all reactions. Reactions with each primer/probe pair and template were performed in triplicate. Following baseline correction, a fluorescence threshold was established and the cycle when this threshold was crossed (C_t_) was determined for each reaction. To control for variability in RNA quantity, the normalized value, ΔC_t,_ for each sample was calculated using the formula ΔC_t_  =  C_t(SUR)_ – C_t(Hprt)_ Relative mRNA expression is reported as 2^−ΔCt^* 1000 for atrial and ventricular tissue experiments. The fold change in expression in response to drug treatment is reported as 2^−ΔΔCt^, where ΔΔC_t_  =  ΔC_t__(drug)_ − ΔC_t__(no treatment)_.

### DNA Methylation Analysis

Genomic DNA was isolated from HL-1 cell lines, atrial or ventricular tissue using DNeasy kit (Qiagen, Inc.) following the manufacturer’s protocols. DNA methylation was assessed in two ways–EpiTect® Methyl qPCR Assay (SABiosciences) and by bisulfite sequencing [37].

For the first method, genomic DNA (400 ng) was digested for 6 hours with 1) no restriction enzyme, 2) methylation-sensitive restriction enzyme, 3) methylation-insensitive restriction enzyme, and 4) both restriction enzymes. Following heat inactivation, 10 ng of treated DNA was amplified using primers specific to CpG islands of the SUR1 (cat# MePM09623-2A) or SUR2 (custom designed: Forward 5′-TGGGGTGCCTGCAGTTTCC; Reverse 5′-GATCTCTCTGTAGCAAGCC) gene with SYBR green for monitoring reaction progress. Data was analyzed following the manufacturer’s design.

For the second method, genomic DNA (2 µg) was treated with bisulfite (EZ DNA Methylation™ Kit, Zymo Research) to convert unmethylated cytosine residues to thymidine. 50 ng of bisulfite converted DNA was used as a template for PCR amplification using ZymoTaq™ DNA Polymerase (Zymo Research) of the −158–+71 region of the SUR1 (Abcc8) and −159–+68 of the SUR2 (Abcc9) genes (relative to the start codon +1) using the following primers:

SUR1 Forward: 5′ GTTTTATAAGAGTAGTTGGAAGG 3′

SUR1 Reverse: 5′ TTATTAAAAACACCTTAATCCACCC 3′

SUR2 Forward: 5′ GGTGTTTGTAGTTTTTTGTTAGGG 3′

SUR2 Reverse: 5′ ACAACTTACAACCAATAACTCCTCAA 3′

Resultant PCR products were directly sequenced (Roche 454) with a minimum of 2274 and maximum of 12598 sequences per PCR product (Research and Testing Laboratory, Lubbock, TX). Additional CpG dinucleotides in the “CpG shore” corresponding to 1600 bp upstream of the CpG islands were also analyzed by conventional Sanger sequencing of the PCR product directly or following insertion into the pJET2.1 vector (Fermentas). In all cases, sequence methylation was analyzed using the BISMA software [38].

### Data Analysis

Data were analyzed using Microsoft Excel software with SigmaXL statistical add-on package. Results are presented as mean±SEM (standard error of the mean) unless otherwise noted. Statistical tests and p-values are denoted in figure legends where appropriate.

## Results

### Regional SUR Transcription in the Mouse Heart Underlies Chamber-specific K_ATP_ Structure

**Figure 1:**
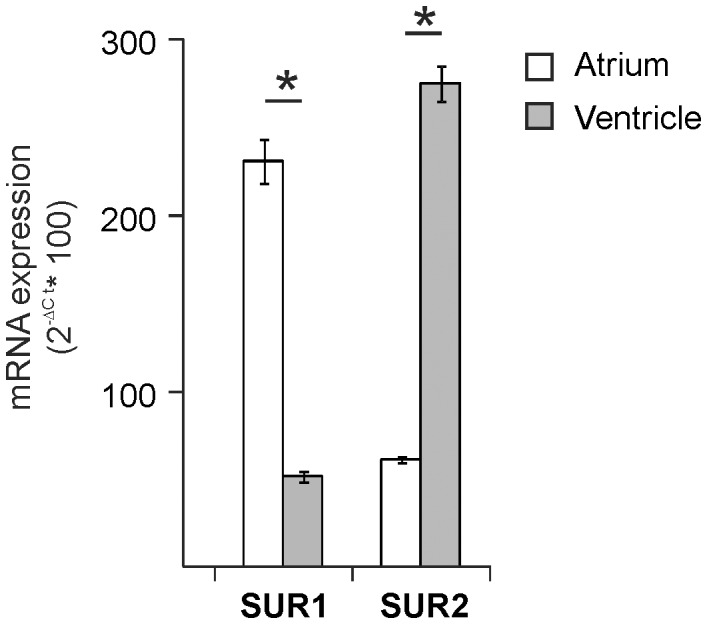
Regional SURx subunit transcription in the mouse heart. Relative mRNA expression of SUR1 and SUR2 obtained in atrial and ventricular tissue (n = 3 hearts each). Expression (normalized to *Hprt*) was assessed by quantitative RT-PCR. Gene-specific Taqman primer and probes were obtained from Applied Biosystems. SUR1 expression was significantly elevated in atrial tissue compared with ventricle, while the opposite distribution was observed for SUR2 (*P<0.001, t-test).

In the cardiovascular system, K_ATP_ channels are formed from the coassembly of Kir6.1 or Kir6.2 with SUR1, SUR2A, or SUR2B [39]. We have recently discovered that sarcolemmal K_ATP_ channels in atrial and ventricular myocytes are distinct–atrial K_ATP_ require SUR1 and ventricular K_ATP_ require SUR2A [29], [30]. Because any combination of K_ATP_ subunits can form when subunits are expressed in heterologous cell expression systems [6], [7], [21]–[28], we postulated that differential subunit mRNA transcription underlies the chamber-specific channel structure. Consistent with this hypothesis, SUR1 mRNA expression is significantly greater in atrial compared with ventricular extracts, while SUR2A shows the opposite distribution (**Fig. 1**). Little is known about the regulation of K_ATP_ channel subunit expression in the heart. To begin to examine this question, we compared and contrasted the genomic sequences immediately upstream and including the first exon of the SUR1 (*Abcc8;* Ch7:53,435,172-53,435,403) and SUR2 (*Abcc9;* Ch6:142,650,684-142,650,794) genes to identify factors that potentially regulate subunit expression.

### CpG Islands in the SUR1 and SUR2 Genes

**Figure 2:**
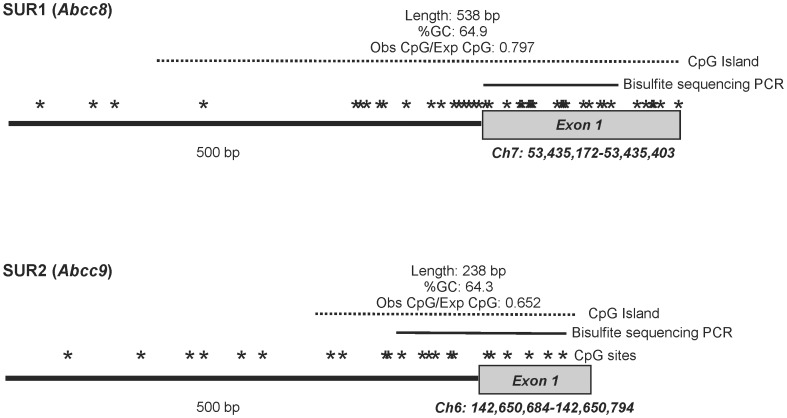
CpG islands in the SUR1 (Abcc8) and SUR2 (Abcc9) genes. Cartoon illustrating the first exon and proximal upstream region of the SUR1 (*top*) and SUR2 (*bottom*) genes are shown. Sequence analysis with CpG Island Searcher software [40], reveals CpG islands in both SUR1 and SUR2 genes, that are at least 200 bp in length, with >55% GC content and an observed:expected CpG ratio >0.65. CpG dinucleotides (39 in SUR1 and 16 in SUR2) are marked with an asterisk. The region analyzed in bisulfite sequencing is also marked.

Cursory examination of the SUR1 and SUR2 upstream sequences suggested a concentration of cytosine-guanosine dinucleotides clustered near the transcription start site of each gene. Using the CpG Island Searcher software [40], we analyzed these sequences to determine whether the apparent concentration of CG dinucleotides in the proximal upstream sequences of SUR1 and SUR2 constituted CpG islands, which are commonly associated with the regulation of mammalian gene expression by DNA methylation [35]. Indeed, the regions were detected as CpG islands of at least 200 bp in length, with >55% GC content and an observed:expected CpG ratio >0.65 (**Fig. 2**).

### SURx Expression and Evidence for a Role for DNA Methylation in HL-1 cells

**Figure 3:**
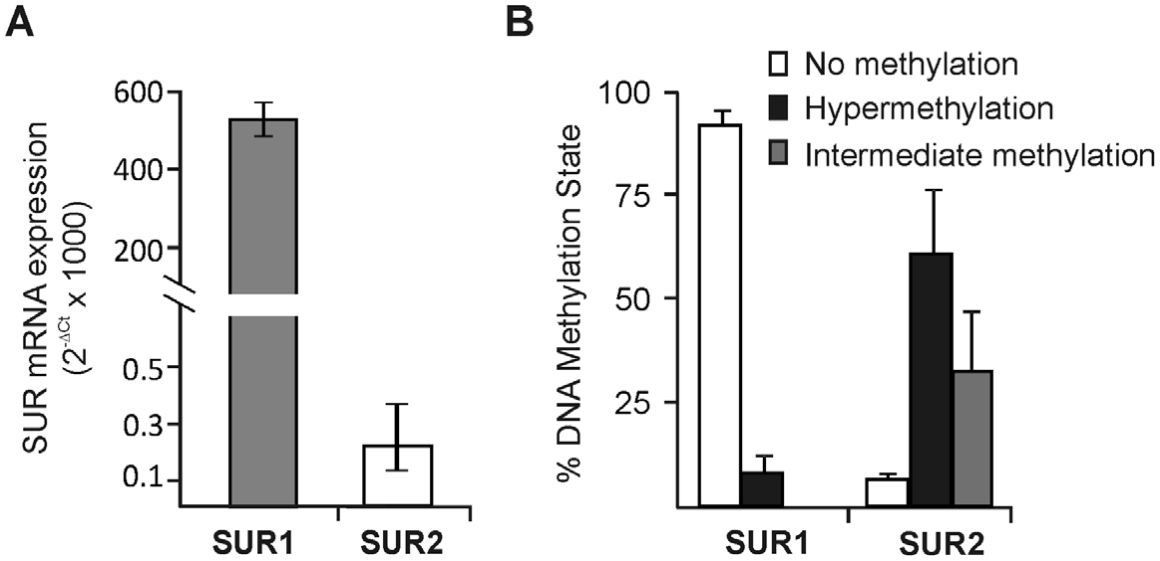
SUR1 and SUR2 expression in HL-1 cells and correlation with CpG island methylation. (A) Relative mRNA expression of SUR1 and SUR2 in HL-1 cells (n = 3). As in the atrium, SUR1 expression is markedly greater than SUR2. (B) The relative absence of SUR2 expression in HL-1 cells is correlated with CpG island methylation. Shown are summary results of methylation state determined using the EpiTect assay (SABiosciences) using primers specific for SUR1 CpG island (cat # MePM09623-2A) and custom primers (see methods for sequences) that amplify the SUR2 CpG island sequence.

**Figure 4:**
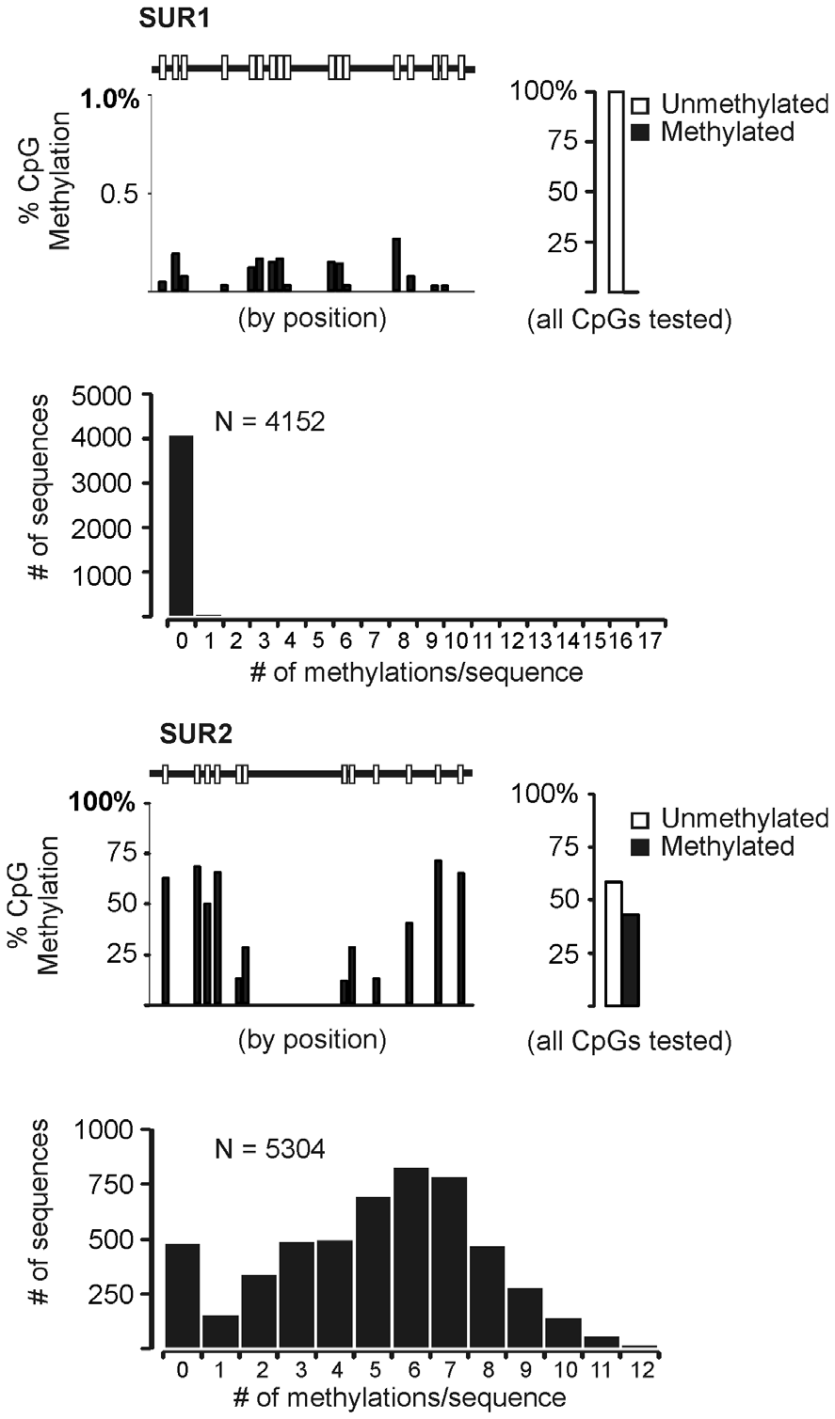
CpG methylation in the CpG island of SUR1 and SUR2 gene in HL-1 **cells.** Shown are data obtained from bisulfite sequencing of the (A) SUR1 and (B) SUR2 sequences. 4152 and 5304 individual sequences were analyzed for SUR1 and SUR2, respectively. At the top, right of each panel the fraction of sequences that are methylated are denoted by position. At top left, the fraction of methylated residues at all CpGs tested (independent of position) is given. At the bottom of each panel, histograms illustrate the number of methylation events registered per sequence.

In order to test the hypothesis that DNA methylation regulates the chamber specific expression of SUR1 and SUR2, we examined subunit expression in a model cell system of the murine atrial myocardium–HL-1 cells derived from mouse atrial myocyte tumors [41]. These cells have been shown to express many cardiac proteins [41] and display a distribution of SUR1 and SUR2 mRNA similar (SUR1>SUR2) to native atrial tissue (**Fig. 3A**). DNA methylation is expected to silence gene expression while the absence of methylation promotes gene transcription. Assessment of the genomic DNA methylation using the EpiTect Methyl qPCR system (SABiosciences) indicates that the SUR2 CpG island exists principally in a intermediate (33±15%) or hypermethylated (61±16%) state while the SUR1 CpG island is nearly completely unmethylated (92±4%) (**Fig. 3B**). To further examine this, we analyzed DNA methylation status at the single nucleotide level using bisulfite sequencing. Following bisulfite conversion and desulfonation of HL-1 cell genomic DNA, we amplified portions of the CpG islands of both SUR1 and SUR2 genes and determined the sequences of the PCR products directly using 454 pyrosequencing technology (Research and Testing Laboratory, Lubbock, TX). In agreement with the results of the Epitect Methyl qPCR assay, we observed marked CpG methylation of the SUR2 (57.6% of all CpGs analyzed), but not SUR1 (0.14% of all CpGs analyzed) (**Fig. 4**). Moreover, of the 5,304 SUR2 sequences analyzed, only 488 (9.2%) exhibited no methylation events, while 4,082 (98.3%) of the 4,152 SUR1 sequences analyzed were unmethylated, with the remainder showing just one methylated CpG.

**Figure 5:**
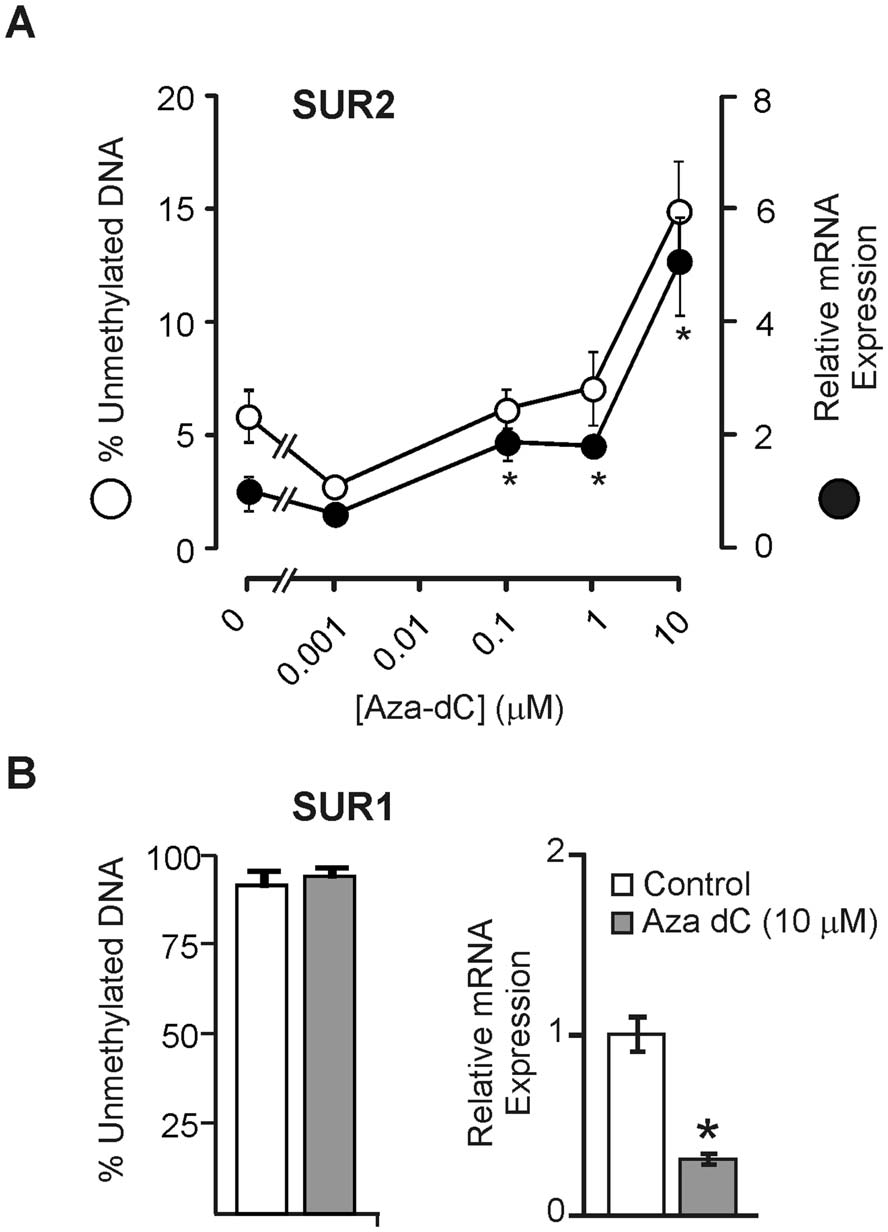
Treatment with 5′-Aza-2′-deoxycitidine (Aza-dC) causes CpG island demethylation and increased expression of SUR2 in HL-1 **cells.** (A) Unmethylated fraction SUR2 CpG island (○) and relative SUR2 mRNA expression (•) at varying doses of Aza-dC. (B) Unmethylated SUR1 CpG island fraction and SUR1 subunit mRNA expression before and after treatment with 10 µM Aza-dC. Taken together, these results indicate that CpG methylation suppresses SURx expression, but that other indirect factors are also likely involved.

Taken together, the data support the conclusion that methylation of the SUR1 and SUR2 promoter can regulate the transcription of the SUR1 and SUR 2 genes. Demethylation of HL-1 genomic DNA would be expected to cause an increase in SUR2 expression with little change or no change in SUR1 expression. To test this, HL-1 cells were treated with 5-aza-2′-deoxycytidine (Aza-dC) to inhibit DNA methylation [42]. Treatment with 10 µM Aza-dC caused a modest (from 5.8±1.1% to 14.9±2.3%, n = 7) but significant shift of the unmethylated fraction of SUR2 CpG island and this was accompanied by a roughly 7-fold increase in SUR2 mRNA expression (**Fig. 5A**), indicating that relief of DNA methylation permits expression of the SUR2 subunit. Interestingly, however, the dose response appears to be biphasic, such that even at a dose (0.1 µM) where there is no apparent change in the overall unmethylated fraction of the SUR2 CpG island, there remains a small (2-fold) increase in SUR2 mRNA expression. Similarly, we observe a small but significant decrease in SUR1 expression with Aza-dC treatment, despite the fact that its CpG island is already principally unmethylated (**Fig. 5B**). This suggests that Aza-dC may actually have multiple effects. For example, recent reports have demonstrated that Aza-dC treatment can reactivate gene expression through ATM- and Rad3-related signaling cascade activation of p53/p21^Waf1/Cip1^ [43]or by degradation of pRb pocket proteins [44] Taken together, these observations suggest that demethylation of the SUR2 CpG alone is not the only factor controlling subunit expression by Aza-dC, but that Aza-dC may also activate additional transcriptional control programs that also regulate SURx subunit expression.

### DNA Methylation and SURx Expression in Atrial and Ventricular Cells

**Figure 6:**
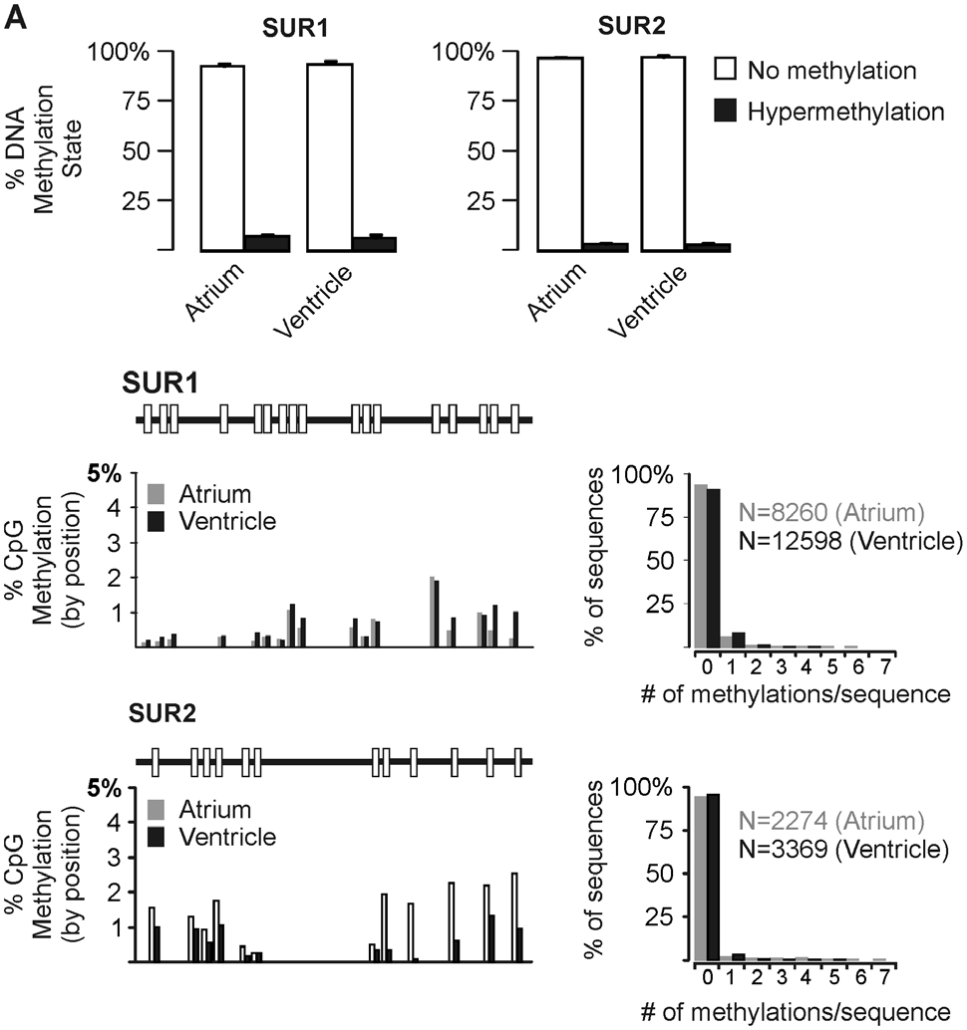
Chamber-specific SUR1 and SUR2 expression does not correlate with CpG island methylation. Total CpG island methylation of SUR1 and SUR2 as measured using either (A) EpiTect assay or (B) bisulfite sequencing. Using either method, SUR1 and SUR2 CpG islands were predominantly unmethylated in both atrial and ventricular genomic DNA. In panel B, CpG methylation is analyzed in a position-specific (left) and whole sequence-specific manner (right). No CpG residue was methylated in more than about 2% of all sequences tested.

The above results suggest a correlation between CpG island methylation and SURx expression in the HL-1 model cell system. To determine whether this mechanism contributes to the chamber-specific distribution of SUR1 and SUR2 in the mouse heart, we analyzed the CpG island methylation in genomic DNA isolated from atrial or ventricular myocytes, using either the Epitect Methyl qPCR assay or bisulfite sequencing analysis (**Fig. 6**). As expected for actively transcribed genes, the SUR1 and SUR2 CpG islands are principally unmethylated in atrial and ventricular genomic DNA, respectively. However, the SUR1 and SUR2 CpG islands are also unmethylated in the tissues where they are not expressed–i.e. SUR1 is unmethylated in ventricle and SUR2 is unmethylated in atrium.

**Figure 7:**
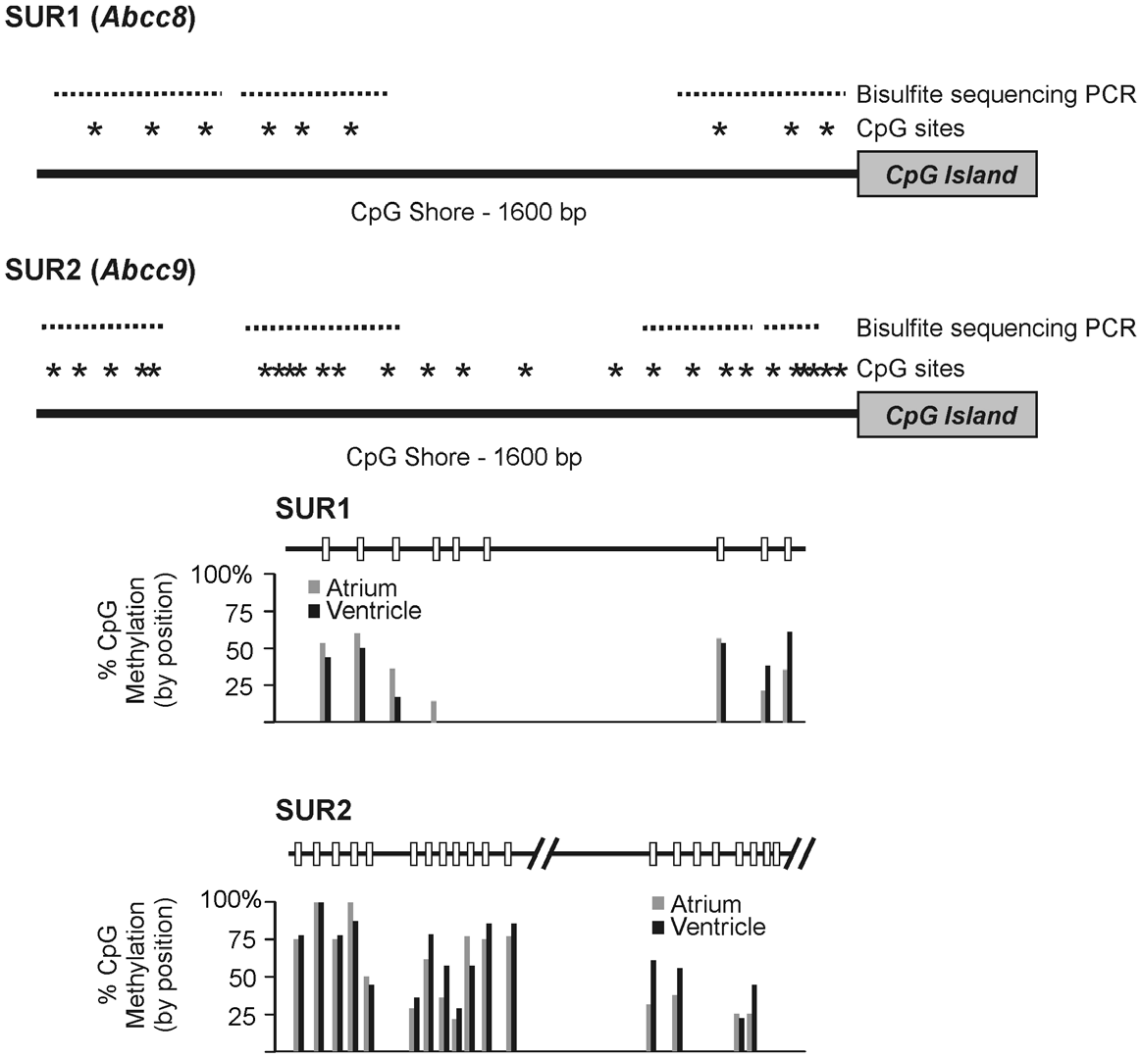
Chamber-specific SUR1 and SUR2 expression does not correlate with CpG shore methylation. (**A**) Cartoon illustrating the CpG shore regions comprised of ∼1600 bp upstream of the CpG islands of the SUR1 and SUR2 genes. CpG dinucleotides (*asterisks*) and specific regions analyzed by bisulfite sequencing are illustrated (*dashed lines*). (**B**) Percent methylation at each of the residues tested is shown. In contrast to the CpG island where no CpG tested exhibited more than 5% methylation in atrial or ventricular DNA, we observed methylation in excess of 50% at many residues in the CpG shore. As in the island, there were no apparent differences in methylation status at any of the residues tested in atrial or ventricular genomic DNA.

Recent studies have suggested that methylation of the CpG “shores”–extending approximately 2 kB upstream of a CpG island–rather than islands might be important in determining tissue specific expression [45], [46]. We performed additional bisulfite sequencing experiments to analyze methylation of CpG shore residues of atrial and ventricular genomic DNA. We limited our analysis to the 1600 bp upstream of the CpG islands containing 9 and 26 CpG dinucleotides in the SUR1 and SUR2 genes, respectively (**Fig. 7**). Unlike the CpG islands where CpG residues were predominantly unmethylated in atrial and ventricular genomic DNA, many but not all of the CpG residues in the shore regions were predominantly methylated. Some residues were essentially unmethylated, however, as in the CpG island, the methylation pattern was similar between atrial and ventricular samples.

Taken together these data indicate that DNA methylation is a potential regulator of SUR1 and SUR2 expression in cardiac myocytes, but it does not appear to regulate the normal atrio-ventricular gradient of SURx expression that underlies chamber-specific K_ATP_ composition. This conclusion is also in general agreement with experiments in HL-1 cells, where factors in addition to CpG demethylation likely influence SUR1 and SUR2 expression.

## Discussion

### Chamber-specific SURx Expression Determines Atrial and Ventricular K_ATP_ Channel Structure

Tissue-specific composition is a key feature of the K_ATP_ channel. We have previously shown that sarcolemmal K_ATP_ channels in atrial and ventricular myocytes are structurally distinct [29], [30]. Atrial K_ATP_, composed of SUR1 and Kir6.2, exhibit characteristic sensitivity to diazoxide and are completely absent in SUR1^−/−^ animals. Conversely, ventricular K_ATP_ channels, made up of SUR2A and Kir6.2, are sensitive to pinacidil and essentially absent in animals lacking the full-length SUR2A subunit [47]. Both atrial and ventricular K_ATP_ are abolished in atrial and ventricular myocytes in Kir6.2^−/−^ mice [12], [13], [48]. Recent evidence indicates yet another subunit combination in cells of the conduction system [31]. Different subunit combinations give rise to channels with distinct functional and pharmacological properties. The functional significance of the chamber-specific K_ATP_ structure in the heart remains unclear, however, it is likely that different subunit composition contributes to the dynamic regulation of pancreatic β-cell (SUR1+ Kir6.2) channels by blood glucose, while ventricular K_ATP_ appears to be less sensitive to modest changes in cell metabolism. The molecular underpinnings of tissue-specific structure have not been well characterized. In heterologous cell systems, in which K_ATP_ channel subunits are exogenously expressed, it has been shown that all subunit combinations can and do occur [6], [7], [21]–[28]. Thus, it is likely that tissue-specific expression is determined by subunit expression and the distribution of SUR1 and SUR2 mRNA indicates that K_ATP_ structure is defined by chamber-specific subunit transcription (**Fig. 1**).

### Methylation-dependent Regulation of SUR1 and SUR2 Expression

Initially described as inheritable factors influencing expression that do not require changes in nucleotides, it is increasingly recognized that epigenetic chromatin modifications can play a role in determining when and where genes are expressed. Methylation of genomic DNA is one of the known and well characterized epigenetic modifications that influence gene expression [35]. CpG islands are targets for DNA methylation and based on our identification of CpG islands in the promoter region of both SUR1 and SUR2 genes (**Fig. 2**), we hypothesized that DNA methylation might be a regulator of SUR1 and SUR2 expression in cardiac myocytes. The data indicate that this is the case in immortalized HL-1 cells that are derived from atrial myocytes (**Figs. 3** **,** **4** **,** **5**); however, in adult murine atrial and ventricular tissue, the evidence does not support a role for DNA methylation in regulating SUR1 or SUR2 expression (**Fig. 6** **–** **7**). Taken together, the data support the model that in some instances (e.g. HL-1 cells), DNA methylation can regulate, in part, SURx subunit expression, while in other cases (e.g. atrial and ventricular myocytes) alternative mechanisms such as tissue specific transcription activator (or repressor) expression seem to determine the distribution of SURx.

### Potential Significance for Methylation-dependent Regulation of SUR1 and SUR2 Expression and Physiological Implications

K_ATP_ channels are key molecular elements that protect the heart during stress [49]. This is the first study to explore the role of DNA methylation in regulating K_ATP_ channel composition. Although DNA methylation does not control the distribution of SUR1 and SUR2 in the atrial and ventricular myocytes, the evidence supports the notion that under some circumstances (e.g. HL-1 cells) CpG methylation can act to silence SURx gene expression. This raises the possibility that aberrant (or regulated) DNA methylation could alter cardiac K_ATP_ subunit expression, affecting the cardiac response to stress. In support of such a notion, TNFα a cytokine which is elevated in the failing heart, has recently been shown to induce a decrease in SERCA2 expression that correlates with promoter methylation [50]. Environmental factors, such as hypoxia, toxins or age, could cause similar changes in methylation and expression [51], [52], with alterations in K_ATP_ current density being either detrimental or beneficial. For example, it has recently been suggested that exercise training confers a significant benefit on cardiovascular function, which correlates with an increase in cardiac SUR2A expression [53]. Conversely, an increase in SUR1 expression in atrial myocytes from hypertensive animals increases the potential for developing atrial fibrillation [54].

It remains unknown why there is a discrepancy between the methylation pattern in HL-1 cells and native tissue. High levels of de novo methylation in cell lines has been previously reported, suggesting that genes that are either unnecessary for or detrimental to cell survival under typical culture conditions are silenced by methylation [55]. Given that channel activation is generally considered to limit cell excitability, reduce Ca^2+^ entry, and prevent cell death during metabolic stress, it is not clear how methylation and suppression of K_ATP_ gene expression would promote cell survival. Rather, since K_ATP_ channels are generally inactive under normal metabolic conditions, it is possible that K_ATP_ channel activity is unnecessary for cell growth and thus silenced. However, it is unclear why this should be specifically applicable to the SUR2 gene, since the SUR1 gene is unmethylated and expressed.

### Conclusion

In summary, the results presented in this study indicate for the first time that K_ATP_ channel subunit expression can be regulated by an epigenetic mechanism, namely CpG methylation. DNA methylation appears to be an important silencing mechanism of SUR2 expression in the transformed HL-1 cells. Although this mechanism does not seem to contribute to determining the chamber-specific structure of sarcolemmal K_ATP_ channels as we hypothesized, this finding raises the possibility that aberrant DNA methylation in disease states or as a result of environmental exposures could alter K_ATP_ channel composition or density, affecting the ability of the heart to respond to metabolic stressors like ischemia.
